# Identification of Biomarkers in Neuropsychiatric Disorders Based on Systems Biology and Epigenetics

**DOI:** 10.3389/fgene.2019.00985

**Published:** 2019-10-11

**Authors:** Jacob Peedicayil

**Affiliations:** Department of Pharmacology and Clinical Pharmacology, Christian Medical College, Vellore, India

**Keywords:** biomarkers, epigenetic, network biology, neuropsychiatric disorders, systems biology

## Abstract

Clinically useful biomarkers are available for some neuropsychiatric disorders like fragile X syndrome, Rett syndrome, and Huntington’s disease. Despite many decades of research on the pathogenesis of neuropsychiatric disorders like schizophrenia (SZ), bipolar disorder (BD), and major depressive disorder (MDD), the exact pathogenesis of these disorders remains unclear, and there are no clinically useful biomarkers for these disorders. However, there is increasing evidence that abnormal epigenetic mechanisms of gene expression contribute to the pathogenesis of SZ, BD, and MDD. Both systems (or network) biology and epigenetics (a component of systems biology) attempt to make sense of biological systems that are highly dynamic and multi-compartmental. This article suggests that systems biology, emphasizing the epigenetic component of systems biology, could help identify clinically useful biomarkers in neuropsychiatric disorders like SZ, BD, and MDD.

## Introduction

A biomarker, a short form for biological marker, has been defined as a feature that is objectively quantified and evaluated as an indicator of normal biological processes, pathological processes, or a pharmacological response to a therapeutic intervention (Biomarkers Definitions Working Group, 2001). In addition, there is another type of biomarker termed physiological biomarkers, which are indicators of the body’s physiological functioning, such as heart rate, breathing rate, and the rate and pitch of speech (Adams et al., 2017). Biomarkers have many uses such as in the evaluation of drug effects in preclinical and clinical drug trials, in the diagnosis of patients with a disease, for staging diseases, as indicators of disease prognosis, and for predicting and monitoring clinical response to an intervention (Biomarkers Definitions Working Group, 2001).

Although clinically useful biomarkers are available for several medical disorders, as well as neuropsychiatric disorders like fragile X syndrome (FXS), Huntington’s disease (HD), and Rett syndrome (RTT), there are at present none available for neuropsychiatric disorders like schizophrenia (SZ), bipolar disorder (BD), and major depressive disorder (MDD) (Davis et al., 2015; Kruse et al., 2017). The current article discusses the possible use of systems biology in the identification of biomarkers for neuropsychiatric disorders like SZ, BD, and MDD. Since epigenetics, like systems biology, attempts to make sense of biological systems that are highly dynamic and multi-compartmental (Housely et al., 2015), among the different components of systems biology, this article gives emphasis to the epigenetic component of systems biology. Another reason for the emphasis on the epigenetic component of systems biology is that there is increasing evidence that abnormal epigenetic mechanisms of gene expression play a crucial role in the pathogenesis of neuropsychiatric disorders like SZ, BD, and MDD (Peedicayil and Grayson, 2018a; Peedicayil and Grayson, 2018b).

### Types of Neuropsychiatric Disorders

Neuropsychiatric disorders comprise a wide range of disorders. They include neurological/neurosurgical disorders and psychiatric disorders. Although neurological and psychiatric disorders differ from each other in many ways, they are also similar to each other, and in some ways are like two sides of the same coin (Peedicayil et al., 2016a). It has been suggested that neurology and psychiatry are two sub-specialties of neuropsychiatry, which is part of the broader specialty of neurosciences (Peedicayil et al., 2016a).

### Reductionism in Neuropsychiatric Disorders

Neuropsychiatric disorders are complex, heterogeneous disorders resulting from the interaction of various factors including genetic, epigenetic, neurobiological, and environmental factors (Lin and Huang, 2016). Can complex biological phenomena like neuropsychiatric symptoms like hallucinations, delusions, disorganized thinking, and mood swings be reduced to specific genes? Noted biologists like Lewontin (1991) and Rose (1995) suggest that psychiatric disorders cannot be reduced to specific genes. Strohman (1997) suggests that epigenetic defects underlying common disorders cannot be identified. He suggests that in future, genetic testing will be restricted to the rare disorders that show Mendelian inheritance. More recently, Drayna (2006) suggests that although simple human behaviors instinctive and crucial to survival and reproduction may be reducible to a set of genes, more generally, human behavior cannot be viewed as a product of a set of genes. Gold (2009) opines that research on the biology of psychiatric disorders is a gamble, like all scientific research. His answer to the question whether reduction is possible in psychiatry is that we will only know after the science has been done.

These workers’ ideas appear to contradict those of Francis Crick (1966) who in *Of Molecules and Men* suggests that the ultimate aim of the modern movement of biology is to explain all biology in terms of physics and chemistry. Even Crick’s colleague James Watson (2003) felt that the secret of life lies in the sequence of bases in DNA. Watson felt that there is no need to invoke vitalism (the theory that the origin and phenomena of life are determined by a force or principle distinct from purely physical or chemical forces) to explain life, and, instead, life can be explained by physicochemical processes. However, both Watson and Crick have been criticized by others (Lewontin, 1991; Strohman, 1997) for their extreme reductionist views.

It is significant that despite a lot of research spread across about a century, there is no conclusive and unambiguous evidence of consistent changes in biochemical (Kruse et al., 2017), neuropathological (Gandal et al., 2018), and neuroimaging studies (Brugger and Howes, 2017) of neuropsychiatric disorders like SZ, BD, and MDD. Presently, the best way to diagnose whether someone has such a disorder or not is to take a good history and conduct a good mental status, neurological, and physical examination (Kruse et al., 2017).

### The Role of Epigenetics in Neuropsychiatric Disorders

A large amount of research on the epigenetics of neuropsychiatric disorders has been conducted over the past few decades. The data gathered so far have shown some interesting disparities (Peedicayil et al., 2016b): the role of epigenetics in the development of neuropsychiatric disorders with a major neurological component like FXS, HD, and RTT has been well characterized. However, in neuropsychiatric disorders with a major psychiatric component like SZ, BD, and MDD, the elucidation of the role of epigenetics in the development of disease is proving to be arduous. The reasons suggested for this disparity could be the following (Peedicayil et al., 2016b): the investigation of the role of epigenetics in neuropsychiatric disorders with a major neurological component started earlier; neuropsychiatric disorders with a greater neurological component are biologically less complex; there is a greater role played by environmental factors in the development of neuropsychiatric disorders with a greater psychiatric component. These three explanations could be related to each other (Peedicayil et al., 2016b).

### Difficulties in Identifying Biomarkers in Neuropsychiatric Disorders

There are several difficulties in finding clinically useful biomarkers for many neuropsychiatric disorders. Liu (2016) has elegantly discussed these problems: First, for many neuropsychiatric disorders, we have a limited knowledge of the pathogenesis of the disorder, and the pathogenesis involves genetic, epigenetic, and environmental factors. Second, many neuropsychiatric disorders have subtypes. Hence, it is difficult to obtain specific, stable, and consistent biomarkers for clinical use. The variation in gene expression between cells, tissues, and patient populations makes identification of biomarkers difficult. Third, the use of the techniques, instruments, and machines for measuring disease parameters are complicated. Additionally, brain tissues are difficult to access, and peripheral tissues have to be used as proxies for brain tissues (Lin and Huang, 2016). Moreover, for many disorders like SZ, BD, and MDD, there are no suitable animal models (Lin and Huang, 2016).

There already are molecular tests for diagnosing some neuropsychiatric disorders. Such neuropsychiatric disorders have a greater neurological than a psychiatric component. They include RTT (Eyal et al., 2019), HD (Nance, 2017), and FXS (Wattendorf and Muenke, 2005). It must be noted that the molecular tests for these disorders involve genetic rather than epigenetic testing.

For the past several decades, a lot of research has been conducted to determine the genetic basis of neuropsychiatric disorders like SZ, BD, and MDD. Such research has the potential to throw light on the pathogenesis of these disorders and also identify genetic biomarkers for the disorders. Such research includes genetic linkage studies, genetic association studies, and genome-wide association studies (GWAS). So far, no genetic mutation or polymorphism predisposing to such disorders has been conclusively identified (Ebstein, 2018; Peedicayil and Grayson, 2018a; Peedicayil and Grayson, 2018b). In GWAS, several associations have been identified (Ebstein, 2018; Peedicayil and Grayson, 2018a; Peedicayil and Grayson, 2018b). However, association does not imply causation (Altman and Krzywinski, 2015). Research on the epigenetic mechanisms underlying neuropsychiatric disorders like SZ, BD, and MDD has led to several findings (Guidotti et al., 2014; lkegame et al., 2013; Kang et al., 2019; Liu et al., 2018; Mor et al., 2013; Tseng et al., 2014; Ziegler et al., 2016) (**Table 1**). However, these need confirmation and validation. In this context, it has been suggested that it would be a good idea to combine genetic and epigenetic data, as well as other “omic” data in order to distinguish signals from background noise and get a clearer picture about the pathogenesis of these disorders (Califano et al.2012; Feinberg, 2018; Wang et al., 2018).

**Table 1:** A partial list of epigenetic changes in some neuropsychiatric disorders.

Sl. No.	Epigenetic Change	Disorder	Tissue
1.	Hypermethylated GAD1, RELN genes	SZ, BD	Peripheral blood cells
2.	Hypermethylated BDNF gene	SZ, BD, MDD	Peripheral blood
3.	Elevated miR-382-5p	SZ	Olfactory epithelium
4.	Several miRNAs	SZ	PBMC
5.	FKBP5 hypermethylation	PTSD	Peripheral blood
6.	Hypomethylated MAOA gene	PD	Peripheral blood
7.	Decreased 5-hmC	MDD	Leukocytes

BDNF, Brain-derived neurotrophic factor; BD, Bipolar disorder; GAD1, Glutamic acid decarboxylase1; hmC, Hydroxymethylcytosine; MDD, Major depressive disorder; miRNA, microRNA; PBMC, Peripheral blood mononuclear cells; PD, Panic disorder; PTSD, Post-traumatic stress disorder; SZ, Schizophrenia. References: 1. Guidotti et al. (2014); 2. Ikegame et al. (2013); 3. Mor et al. (2013); 4. Liu et al. (2018); 5. Kang et al. (2019); 6. Ziegler et al. (2016); 7. Tseng et al. (2014).

### Systems (Network) Biology and Neuropsychiatric Disorders

It is becoming increasingly clear that a clear biological function usually cannot be attributed to a single molecule. Instead, most biological traits arise from complex interactions between a cell’s many constituents like DNA, RNA, and small molecules (Barabasi and Oltvai, 2004). A key challenge for biology in this century is to determine the structure and dynamics of the complex intercellular web of interactions contributing to the structure and functioning of a cell. Many types of interaction webs or networks emerge from a sum of these interactions. None of these networks are independent. Instead, they form a “network of networks” that is responsible for the behavior of a cell. A major challenge of contemporary biology is to theoretically and experimentally map out, understand, and model, in quantifiable terms, the topological (structural) and dynamic properties of the various networks that control the behavior of a cell (Barabasi and Oltvai, 2004).

The new area of systems or network biology could provide a solution for this challenge. Systems biology was pioneered by the noted scientist Leroy Hood using the galactose gene regulatory circuit in the budding yeast *Saccharomyces cerevisiae* (Ideker and Hood, 2019). Systems biology regards biology as an information science, and investigates biological systems as a whole, including their interactions with the environment (Wang et al., 2010). It evolved from the field of systems engineering in which a linked collection of component parts constitute a network whose output the engineer wishes to predict. It refers to a comprehensive quantitative analysis of the manner in which all components of a biological system interact functionally over time (Aderem, 2005). Major developments in technology have taken place since the 1980s. They include automated DNA sequencing, microarray analysis, advances in mass spectrometry, next-generation sequencing, and the internet. The knowledge of the complete sequences of genomes, along with technology allowing the monitoring of the flow of information resulting in specific cell functions, enabled systems biology to develop (Aderem, 2005), a discipline that may change the intellectual and experimental landscape on which we stand (Hiesinger and Hassan, 2005).

All systems can be analyzed by defining their static topology (architecture) and their dynamic (time-dependent) response to perturbation (Loscalzo, 2018). Any system of interacting elements can be schematically represented as a network comprising individual elements (nodes) connected by edges. The nature of the edges reflects the degree of complexity of the system. In simple systems, the nodes are linked linearly with a few feedback or feed-forward loops modulating the system in predictable ways. In complex systems, the nodes are linked in complicated non-linear networks. An important property of complex systems is that simplifying their structures by identifying and characterizing their individual nodes or edges or simple sub-structures need not yield a predictable understanding of a system’s behavior. Hence, the system is greater than, or different from, the sum of its individual parts (Loscalzo, 2018).

Systems biology will help us attain a more holistic picture of disease states and could vindicate the reductionist approach to biology (Hiesinger and Hassan, 2005). It will not only facilitate basic biological research but also provide new ways to understand human diseases, identify biomarkers, and develop treatments for diseases (Wang et al., 2015). Moreover, systems biology may help answer questions related to complex organs like the brain, questions which cannot be answered with only the currently available tools of molecular biology and genomics (Villoslada et al., 2009).

### Systems Biology and Biomarkers in Neuropsychiatric Disorders

**Figure 1:**
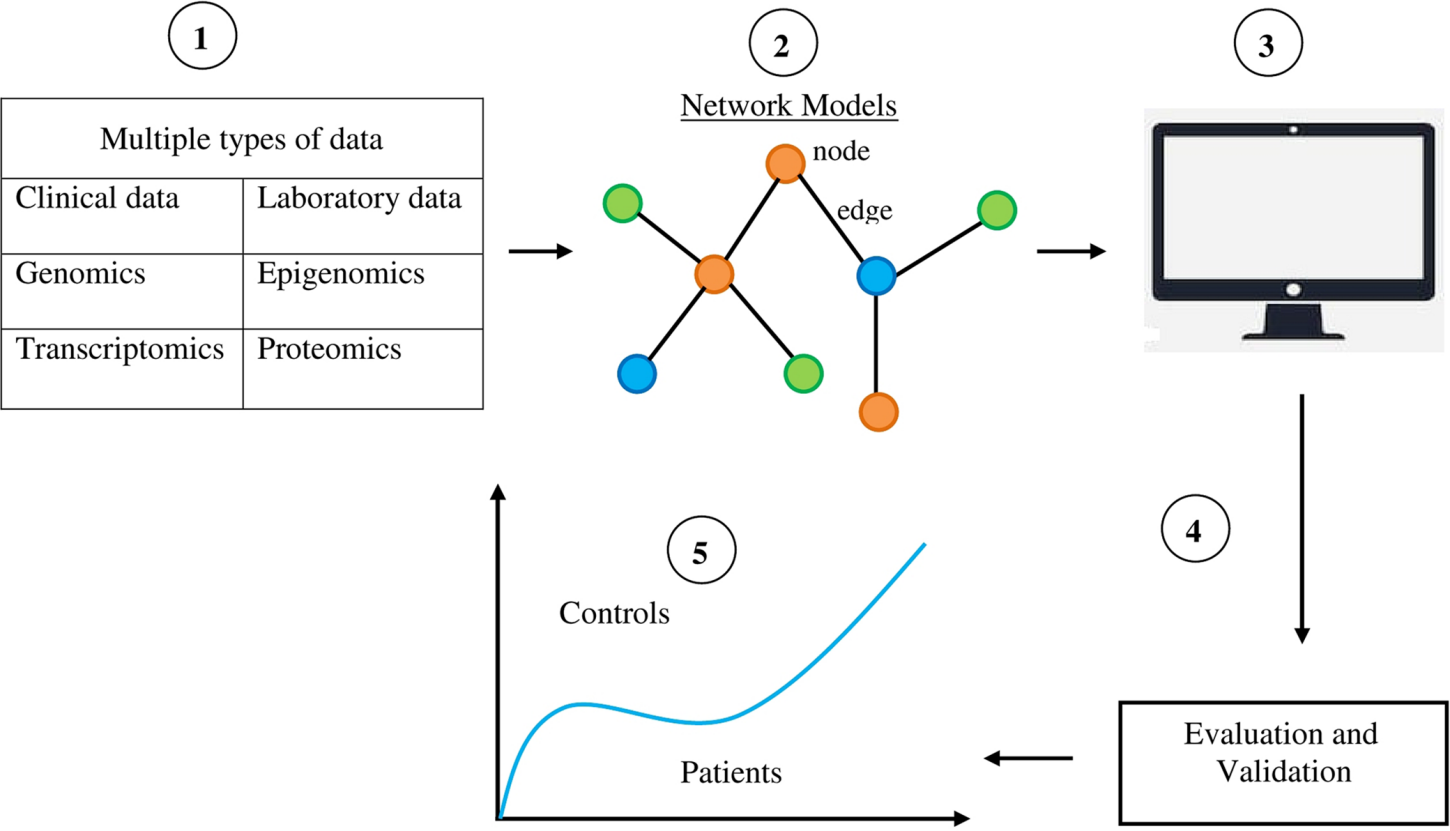
Diagrammatic and simplified representation of the use of systems biology to identify biomarkers for neuropsychiatric disorders. 1) Collection of multiple types of data from patients, thereby providing the resources for the discovery of biomarkers. 2) The data gathered about molecules like DNA, RNA, proteins, and metabolites are organized by a network model. Components of networks like nodes and edges provide the materials for identifying disease biomarkers. 3) Computerized selection is done of specific features and abilities in network components for classifying various phenotypic states. 4) The data obtained are evaluated and validated in order to distinguish signal from noise. 5) Systems biology-based biomarkers are used to distinguish phenotypic states like normal and disease states.

Systems biology could help identify biomarkers for neuropsychiatric disorders (**Figure 1**). As discussed by Lausted et al. (2014), the challenge in identifying biomarkers for complex disorders is to distinguish a small signal from a large amount of noise. The usual approach to blood-based biomarker discovery is to compare molecular profiles of blood samples from normal individuals with those from patients. Inevitably, large numbers of differences are found. However, a lot of these biomarkers is noise (Köhler and Seitz, 2012). A systems approach to biomarkers provides powerful tools for distinguishing signals from noise (Ideker et al., 2011; Lausted et al., 2014). This is because networks provide a distinct and rational framework for describing interactions between genes, RNA, proteins, and metabolites, and organizing the available data simultaneously (Liu, 2016). Molecules interact as a network in performing their functions. The nodes represent these molecules and the edges represent their physical and functional relationships. The network provides a topological representation of a complex system and the data characterize its specific condition by quantitatively measured values of a large number of molecules. Systems biology uses sophisticated computer software “omics”-based discovery tools and advanced computational techniques to understand the behavior of biological systems and identify diagnostic and prognostic biomarkers for complex disorders (Alawieh et al., 2012). A systems biology biomarker differs from traditional individual biomarkers in that a systems biology biomarker is a sub-network comprising two or more differentially expressed components in control samples *versus* disease samples (Wang et al., 2015).

Lausted et al. (2014) suggests that a systems biology approach for the discovery of biomarkers needs to use the following principles: 1) Blood is the ideal tissue/fluid for assessing biomarkers since it bathes all organs and contains secreted or released proteins from all these organs (however, it must be noted that for neuropsychiatric disorders there is a caveat regarding this principle in that the blood–brain barrier does not permit many molecules from crossing). 2) The diagnostic analyses should be conducted in a longitudinal manner so that changes in disease states can be followed. 3) The analyses should be quantitative. 4) Each patient should be his or her own control. 5) Multiple biomarkers should be measured since testing the status of multiple networks within the organ of interest is advantageous and probably needed. 6) Biomarkers may be of different informational types, like mRNAs, miRNAs, proteins, metabolites, and lipids.

In order to overcome the current limitations of systems biology and boost the efficiency of the systems biology approach for identifying biomarkers in neuropsychiatric disorders, researchers are coming up with innovative ideas and solutions like using neuroimaging techniques to study structural brain changes in patients (Frank et al., 2018), using induced pluripotent stem cell technology to model brain disorders (Schadt et al., 2014), and using endophenotyes (measurable components unseen by the unaided eye along the pathway between disease and distal genotype) of diseases (Gottesman and Hanson, 2005).

There is currently a new initiative called “The Psychiatric Cell Map Initiative” which aims to identify the physical and genetic interaction networks of neuropsychiatric disorders, and then using these data to connect genomic data to neuroscience and finally the clinic (Willsey et al., 2018). The initiative will include geneticists, structural biologists, neurobiologists, systems biologists, and clinicians; use many experimental approaches; and create a collaborative team for long-term investigation. Its goal is to determine novel molecular and functional interaction data and pathway-level insights with regard to risk genes. The results of this initiative could have several applications, including identification of clinically useful biomarkers (Willsey et al., 2018).

### Concluding Remarks

Neuropsychiatric disorders appear to be entirely biological: based on the activities of genetic and epigenetic mechanisms of expression of genes in neurons and other types of cells in different parts of the brain. As James Watson (1963) remarked in his 1962 Nobel banquet speech, the day he and Francis Crick discovered the structure of DNA, “they knew a new world had been opened and that an old world that seemed rather mystical was gone.” There is unlikely to be a need to invoke mysticism or vitalism to explain partly or entirely our thoughts and feelings, normal or abnormal. However, due to the inordinate complexity of the brain, it remains to be seen whether neuropsychiatric disorders like SZ, BD, and MDD can be reduced to proteins, amines, or nucleic acids. For several decades, researchers have tried to find proteins and amines as biomarkers for these disorders, with no avail (Kruse et al., 2017). If these disorders could not be reduced to these molecules despite voluminous research, they may not also be reducible to nucleic acids like DNA. Regarding the human brain and mind, “the whole may be greater than the sum of its parts,” a phrase attributed to Aristotle in its original form. Peter Medawar (1984) in *The Limits of Science* states that science can solve questions that come under the realm of science, but may not be able to solve questions that come under the realms of religion and philosophy. I feel that the development of neuropsychiatric disorders like SZ, BD, and MDD comes under the realm of science, and not religion and philosophy, and should be solvable by the methods of science. The methods and techniques of systems biology, incorporating epigenetic and other data, may help identify clinically useful biomarkers for neuropsychiatric disorders.

## Author Contributions

The author confirms being the sole contributor of this work and has approved it for publication.

## Conflict of Interest

The author declares that the research was conducted in the absence of any commercial or financial relationships that could be construed as a potential conflict of interest.
